# The Mosquito Larvicidal Activity of Essential Oils from *Cymbopogon* and *Eucalyptus* Species in Vietnam

**DOI:** 10.3390/insects11020128

**Published:** 2020-02-17

**Authors:** Ho Dung Manh, Do Thi Hue, Nguyen Thi Thanh Hieu, Doan Thi Thanh Tuyen, Ong Thi Tuyet

**Affiliations:** 1 Department of Pharmaceutical Chemistry, Faculty of Pharmacy, Lac Hong University, Bien Hoa, Dong Nai 810000, Vietnam; 2 Department of Physiology, Faculty of Pharmacy, Lac Hong University, Bien Hoa, Dong Nai 810000, Vietnam

**Keywords:** essential oils, *Cymbopogon species*, *Eucalyptus* species, larvicide, *Aedes aegypti*, Vietnam

## Abstract

The larvicidal activity of essential oils (EOs) extracted from *Cymbopogon citratus, Cymbopogon winterianus, Eucalyptus citriodora*, and *Eucalyptus camaldulensis* aromatic plants grown in Vietnam was evaluated on *Aedes aegypti* larvae. The EOs were hydro-distilled in a Clevenger-type apparatus. The EOs were analyzed by gas chromatography–mass spectrometry (GC–MS). The mortality rates obtained from the bioassays were used to calculate the lethal concentrations (LC_50_) of the EOs by the probit analysis method. These essential oils exhibited toxicity to the larvae of *Aedes aegypti*. Results were obtained for *Cymbopogon citratus* (LC_50_ = 120.6 ppm), *Cymbopogon winterianus* (LC_50_ = 38.8 ppm), *Eucalyptus citriodora* (LC_50_ = 104.4 ppm), and *Eucalyptus camaldulensis* (LC_50_ = 33.7 ppm). The essential oils of *Eucalyptus camaldulensis* and *Cymbopogon winterianus* were found to be the most efficient, and their respective values of LC_50_ were 33.7 ppm, 38.8 ppm. In conclusion, this research adds to the growing body of literature on natural larvicides from essential oils against *Aedes aegypti* mosquitoes.

## 1. Introduction

Dengue is the most important vector-borne infectious disease in the world. It is estimated that more than 50 million infections occur and half a million individuals are hospitalized due to dengue hemorrhagic fever each year. The infection is caused by one of four dengue virus serotypes: DENV-1, DENV-2, DENV-3, or DENV-4. [1,2] The *Aedes* mosquito is the primary vector for the transmission of dengue fever. A successful integrated mosquito control strategy includes several tactics, such as removing mosquito breeding sites and using synthetic chemical insecticides (pyrethroids, organophosphates, etc.) to control the spread of adult or larval forms of *Aedes aegypti* [3].

While synthetic chemicals have successfully controlled *Aedes* mosquito populations, the tremendous increase in their use has led to insecticide resistance in mosquito vectors [4] and adverse effects on non-target organisms, including humans [5].

Therefore, developing safe and eco-friendly insecticides is important for public health. In this direction, plant essential oils (EOs) have been shown to be potential alternatives to synthetic insecticides against mosquito vectors [6,7]. Plant-based insecticides are considered environmentally safe and have no or little effects on non-target organisms, in addition to being locally available in many parts of the world affected by mosquito-borne diseases [8,9,10]. Furthermore, EOs containing mixtures of active components act on insects at different target sites or with different modes of action and thus might reduce resistance in mosquito populations [11].

In the present study, we aim to extract four EOs from *Cymbopogon citratus, Cymbopogon winterianus, Eucalyptus citriodora,* and *Eucalyptus camaldulensis* aromatic plants grown in Vietnam and to determine the larvicidal activity of these EOs against *Aedes aegypti* mosquitoes.

## 2. Materials and Methods 

### 2.1. Essential Oil Extraction 

*Cymbopogon citratus, Cymbopogon winterianus, Eucalyptus citriodora,* and *Eucalyptus camaldulensis* leaves were collected in several locations in Vietnam in July 2018 (Table 1). Voucher specimens (1801, 1802, 1901, and 1902, respectively) have been deposited in the Centre of Research and Applied Technology, Lac Hong University, Vietnam.

Fresh plant leaves (500 g) were submitted to hydrodistillation for 2 h using a modified Clevenger-type apparatus. The essential oils were collected, dried with anhydrous sodium sulfate, and stored in airtight containers before analysis by GC–MS. The yields were determined based on the fresh weight of the leaf plant materials. 

### 2.2. Determination of Major Constituents

The analysis of all essential oils was performed using a gas chromatography–mass spectrometry (GC–MS) system consisting of an Agilent 6890N gas chromatograph coupled to a 5973 mass spectrometer. The chromatographic separation was achieved on an HP-5MS capillary column (30 m × 0.25 mm i.d. × 0.25 μm film thickness). The initial column temperature of 50 °C was held for 2 min and increased by 2 °C/min to 80 °C; then the temperature was raised to 150 °C at 5 °C/min, increased to 200 °C at 10 °C/ min, and increased to 300 °C at 20 °C/ min. The temperature was then held there for 5 min. Helium was used as the carrier gas at a flow rate of 1 mL/min. The injector temperature was maintained at 250 °C. The diluted samples (1:40 v/v in n-hexane) were injected at 1 μL in splitless mode. Each component was identified by using its relative retention index alongside the Wiley registry of mass spectral library and NIST MS Search. The percentage composition was calculated by integrating the peak areas of the spectrograms. 

### 2.3. Mosquito Larvae

The colony of *Aedes aegypti* used in this study was reared in the Department of Parasitology, Faculty of Pharmacy, Lac Hong University. Adults were maintained in cages (30 cm × 20 cm × 20 cm) at a temperature of 27 ± 3 °C, with 70% ± 5% relative humidity under a 16:8 h light/dark cycle. Mosquitoes were maintained on a 10% sucrose solution with the blood of live mice, whereas larvae were reared in plastic trays and fed with Wiskcat food (for cats). The larvae between the third and fourth stages of *Aedes aegypti* served as the test organisms.

### 2.4. Larvicidal Assay

The larvicidal bioassay was carried out according to the recommendations of WHO [12]. The essential oils were serially diluted in ethanol to obtain the stock solutions in different concentrations. Twenty-five 3rd or 4th instar larvae were introduced into cups containing 100 mL of distilled water. Damaged or unhealthy larvae were replaced. For each concentration, we performed tests three times. Control tests were performed simultaneously with 1 mL of alcohol in distilled water. The test containers were kept at 25–28 °C with a photoperiod of a 12:12 h light/dark cycle. Larval mortality was recorded after 24 h exposure.

### 2.5. Data analysis

The 24-h mortality and test concentration data were subjected to probit analysis using the SPSS (IBM, version 22.0, Armonk, NY, USA) software program. The probit-log(concentration) regression model was used to calculate LC_50_ values, 95% fiducial limits, slopes, standard errors (SE), chi-square (χ^2^) value, and degrees of freedom. 

## 3. Results and Discussion

### 3.1. Yield of Essential Oils

Table 1 shows the yields of the EOs obtained by a simple hydrodistillation of *Cymbopogon citratus, Cymbopogon winterianus, Eucalyptus citriodora,* and *Eucalyptus camaldulensis* leaves. The highest oil yield was 2.10%, obtained from *Eucalyptus citriodora*, while the lowest oil yield was 0.33%, obtained from *Cymbopogon citratus* (Table 1).

### 3.2. Chemical Composition of Essential Oils

Table 2 shows the major constituents of the EOs. Four constituents, which accounted for 97.4% of the *Cymbopogon citratus* essential oil, were geranial (49.3%), neral (36.2%), β-myrcene (7.8%), and geraniol (4.1%). The high percentage of citral (85.5 %), which is a mixture of the two geometric isomers geranial and neral, is in good agreement with previous studies [13,14,15]. While *Cymbopogon citratus* is known for its high content of citral, *Cymbopogon winterianus* is known to possess an oil rich in some monoterpenoids, such as citronellal, citronellol, geraniol, and elemol [16,17,18]. In this study, the essential oil of *Cymbopogon winterianus*, also known as citronella oil, mainly consists of elemol (29.5%), citronellal (23.5 %), citronellol (7.5%), and geraniol (6.1%). Citronella oil is well known for its natural mosquito repellent properties and is of great interest in the pharmaceutical, perfumery, and cosmetic industries.

*Eucalyptus*, a member of the family Myrtaceae, is a popular cultivated plant in Vietnam. Among the *Eucalyptus* species, the EO extracted from *Eucalyptus citriodora* usually contains a high content of citronellal. In this study, the major constituents of *Eucalyptus citriodora* oil were citronellal (78.6%), citronellol (12.1%), and isopulegol (7.2%). Plants cultivated in Brazil [19], Argentina [20], and the Democratic Republic of the Congo [21] also produce EOs with a high content of citronellal. While *Eucalyptus citriodora* is known for its high content of citronellal, *Eucalyptus camaldulensis* is known to possess an oil rich in 1,8-cineole. In this study, the major constituents of *Eucalyptus camaldulensis* oil were 1,8-cineol (42.6%), α-pinene (32.1%), and citronellyl acetate (5.6%). *Eucalyptus camaldulensis* oils with a high content of 1,8-cineole were also reported in the Democratic Republic of the Congo [21], Egypt [22], and Tunisia. [23]

### 3.3. Mosquito Larvicidal Activity of Essential Oil

Table 3 shows the mosquito larvicidal activity for *Cymbopogon citratus, Cymbopogon winterianus, Eucalyptus citriodora,* and *Eucalyptus camaldulensis* leaf essential oils. *Cymbopogon citratus* was the least effective EO since it has the highest value of LC_50_. This result is comparable with the results of earlier findings. Sosan et al. [24] and Vera et al. [7] reported that *Cymbopogon citratus* had an LC_50_ of nearly 120 ppm and 123 ppm, compared to 120.6 ppm in the present study. In comparison with *Cymbopogon citratus*, *Cymbopogon winterianus* essential oil has a lower LC_50_ value of 38.8 ppm. Mendonca et al. [25] investigated an ethanol extract of *Cymbopogon winterianus* leaves against *Aedes aegypti* larvae and found a higher LC_50_ value of 98 ppm. This result indicates that *Cymbopogon winterianus* essential oil was more effective against *Aedes aegypti* larvae than its ethanol extract.

In this study, the LC_50_ value of *Eucalyptus citriodora* essential oil was 104.4 ppm. This result is slightly higher compared to the previous findings of Vera et al. [7], where the EO extracted from *Eucalyptus citriodora* grown in Colombia had an LC_50_ value of 70 ppm. In comparison with *Eucalyptus citriodora*, *Eucalyptus camaldulensis* essential oil had a much lower LC_50_ value (33.7 ppm). This low LC_50_ value was in good agreement with previous reports in which *Eucalyptus camaldulensis* essential oil was able to induce 50% mortality at a concentration of around 30.0 µg/mL. [26,27]. In comparison with Bti (LC_50_ = 0.026ppm) [28] and Neem oil formulations (LC_50_ = 1.7 ppm) [29], the LC_50_ of the four essential oils were much higher. Although these EOs show promising activity, the low efficacy of these EOs compared to commercial products has limited their application in practice. 

Regarding the mode of action, several mechanisms through which essential oils produce neurotoxic effects have been proposed, such as EOs acting on GABA receptors, octopamine receptors, or acetylcholinesterase [30]. Further, the synergistic effect of the essential oil constituents likely increases their larvicidal activity [31,32,33]. Scalerandi et al. proposed an interesting mode for synergistic effects using the metabolic model [34]. They have shown that while the major component in the mixture is targeted by the detoxification system of the insect, the minor component intoxicates the insect. As a result, the minor component will be more toxic than when it was assayed alone since the detoxification system of the insect fights the main component.

## 4. Conclusions

Our findings suggest that all the EOs exhibit toxicity to the larvae of *Aedes aegypti*, especially the essential oils of *Eucalyptus camaldulensis* and *Cympobogon winterianus* leaves, which may be potentially suitable for controlling mosquitoes at the larval stage. However, field evaluations and further research on the EOs’ potential toxicology for non-target organisms is necessary.

## Figures and Tables

**Table 1:** Location of the collected plants, registration number (voucher), and yield of essential oil.

Scientific Name	Location	Voucher No.	Yield% (V/W)
*Cymbopogon citratus*	District 9, Ho Chi Minh	1801	0.33
*Cymbopogon winterianus*	Dau Tieng, Binh Duong	1901	1.30
*Eucalyptus citriodora*	Moc Hoa, Long An	1802	2.10
*Eucalyptus camaldulensis*	Bien Hoa, Dong Nai	1902	0.61

**Table 2:** Chemical constituents of essential oils.

Constituents	% of Oil			
	*Cymbopogon citratus*	*Cymbopogon winterianus*	*Eucalyptus citriodora*	*Eucalyptus camaldulensis*
1,8-Cineol				42.6
Neral	36.2			
Citronellal		23.5	78.6	
Citronellyl acetate		2.9	1.7	5.6
Elemol		29.5		
Germacrene D		2.8		
Germacrene D-4-ol		4.7		
Isomenthone				
Isopulegol			7.2	
Geranial	49.3			
Geraniol	4.1	6.1		
α-Pinene				32.1
α-Terpineol				1.8
Citronellol		7.5	12.1	
β-Elemene		4.4		
β-Myrcene	7.8			
β-Pinene				4.4

**Table 3:** Larvicidal activity of each essential oil (EO) evaluated in larvae of *Aedes aegypti* at 24 h.

Essential Oils	*n*	Slope (±SE)	LC_50_ (ppm) (95% FL)		χ^2^	df
*Cymbopogon citratus*	475	5.33 ± 0.44	120.6	(111.6–130)	25.1	17
*Cymbopogon winterianus*	300	3.57 ± 0.48	38.8	(31.5–45.6)	20.4	10
*Eucalyptus citriodora*	375	5.39 ± 0.53	104.4	(98.1–111.4)	12.0	13
*Eucalyptus camaldulensis*	300	6.53 ± 0.64	33.7	(30.1–36.9)	15.3	10

*n*: represents the number of larvae tested.
